# Anti-Struvite, Antimicrobial, and Anti-Inflammatory Activities of Aqueous and Ethanolic Extracts of *Saussurea costus* (Falc) Lipsch Asteraceae

**DOI:** 10.3390/molecules28020667

**Published:** 2023-01-09

**Authors:** Naima Mammate, Fatima Ezzahra El oumari, Hamada Imtara, Salim Belchkar, Ghita Benjelloun Touimi, Mohammed Al-Zharani, Hassan A. Rudayni, Ashraf Ahmed Qurtam, Mohammed S. Aleissa, Fahd A. Nasr, Omar M. Noman, Tarik Sqalli Houssaini

**Affiliations:** 1 Laboratory of Epidemiology and Research in Health Sciences, Faculty of Medicine and Pharmacy, Dental Medicine, University Sidi Mohammed Ben Abdellah, BP 1893, Km 22, Road of Sidi Harazem, Fez 30070, Morocco; 2 Department of Nephrology, University of Hospital Hassan II, BP 1835, Atlas, Road of Sidi Harazem, Fez 30700, Morocco; 3 Faculty of Arts and Sciences, Arab American University Palestine, Jenin P.O. Box 240, Palestine; 4 Laboratory of Human Pathology Biomedicine and Environment, Faculty of Medicine and Pharmacy of Fez, Sidi Mohammed Ben Abdellah University (USMBA), Fez 30070, Morocco; 5 Biology Department, College of Science, Imam Mohammad Ibn Saud Islamic University (IMSIU), Riyadh 11623, Saudi Arabia; 6 Department of Pharmacognosy, College of Pharmacy, King Saud University, Riyadh 11451, Saudi Arabia

**Keywords:** antibacterial activity, FT-IR, minimum inhibitory concentration, minimal bactericidal concentration, *Saussurea costus* (Falc) Lispich, struvite, anti-inflammatory activity

## Abstract

*Saussurea costus* (Falc) Lipsch is a traditional herb used to treat kidney stone problems because it contains several molecules used to treat this health problem, such as quercitrin. Infectious stones are the most painful of all urinary tract disorders, with ammonium phosphate (struvite) and carbapatite stones being the most common, caused by a bacterial infection with urease activity. These stones are treated with antibiotics, but antibiotic resistance is on the rise. The current study investigated the anti-urolithic activities of *S. costus* aqueous and ethanolic extracts of against struvite crystals synthesized using microscopic crystallization and turbidimetric methods, respectively. The utilized methods indicated that the ethanolic extract of this plant has a significant inhibitory effect on struvite crystallization, with a percentage inhibition of (87.45 ± 1.107) (*p* < 0.001) for a concentration of 1 mg mL^−1^ and a decrease in the number of struvite crystals, reaching values less than 100/mm^3^. For the number of struvite crystals inhibited by cystone, we found a value of 400/mm^3^ and with the aqueous extract we found 700/mm^3^. The antibacterial activity of the plant extracts studied was examined against several urease-producing bacteria, and this activity was evaluated by qualitative and quantitative evaluation methods; the highest minimum inhibitory concentration was seen in the ethanolic extract, with an MIC of 50 mg mL^−1^ for *Staphylococcus aureus* followed by an MIC of 200 mg mL^−1^ for *Klebsiella pneumoniae*. It showed a minimal bactericidal concentration (MBC) against *S. aureus* and *K. pneumoniae* (>50 mg mL^−1^ and >200 mg mL^−1^, respectively). Furthermore, to determine the extract’s anti-inflammatory activity, in vivo anti-inflammatory activity was investigated in rats. The results show that at a dose of 400 mg kg^−1^, the ethanolic extract has a maximum edema inhibition of 66%.

## 1. Introduction

Infectious lithiasis affects women three times more than men and is caused by struvite stones [1], which are composed of Ammonium magnesium phosphate [2]. Struvite stones are classified as infectious lithiasis [3] because their presence indicates the presence of a ureolytic germ that can cause a sufficiently high alkalinity of the urine to cause the simultaneous precipitation of ammonium and magnesium phosphates [4].

Infectious lithiasis mechanisms involving struvite formation necessitate physicochemical conditions found only in urine that are modified by the presence of urease-producing microorganisms such as *Proteus* sp. or *Staphylococcus* sp. [5]. The action of bacterial urease involves the hydrolysis of urea, which results in the release of NH_3_, which hydrolyzes into ammonium, causing a strong alkalinization of urine (pH exceeds 8) [6], resulting in the precipitation of struvite [7]; there are other strains, such as *Escherichia coli*, that are not ureasic but are able to alkalinize urine [3,8]. Antibiotics can be used to treat urinary tract infections [9], but the germs that cause these types of stones are becoming increasingly resistant to them [10], necessitating research into new antimicrobial compounds. Struvite stones cause tissue and cell damage as a result of urease germs [11], which causes inflammation [12]. Inflammation is the body’s defense response to various aggressions that can be infectious in nature, such as renal colic [13]. Inflammation is treated with nonsteroidal anti-inflammatory medications [14], which, while effective, frequently have side effects. Furthermore, herbal medicines are gaining interest and are considered an alternative for the control of bacterial infections accompanied by inflammation [15,16].

**Figure 1:**
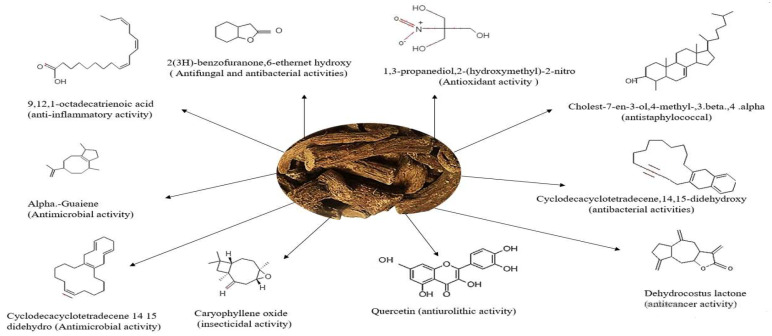
Structures of biologically active organic compounds present in ethanolic and aqueous extracts of *S. costus* (Falc) Lipsch.

*S. costus* is an herbaceous plant of the Asteraceae family native to Asia which has been used to treat various pathologies in traditional medicine [17,18,19]. It contains several molecules, including quercitrin flavonoids and alkaloids (Figure 1), with activities against calcium oxalate type urinary lithiasis [20]. Although it is used as a popular remedy for kidney stones [18], its efficacy and mechanism as an anti-struvite, antimicrobial and anti-inflammatory agent are still unknown. Therefore, in the present study was intended to explore the medicinal properties of the plant *S. costus*, using the ethanolic and aqueous extracts of the plant studied.

The present study aimed to explore the anti-struvite activity of both aqueous and ethanolic extracts of *S. costus* in vitro. This was done using microscopic and turbidimetric crystallization methods. The study also t assessed the antibacterial activity of aqueous and ethanolic extracts of this plant against pathogenic strains, with a focus on antimicrobial tests performed on solid media using the disk diffusion method and the quantitative method to determine minimum inhibitory concentrations (MIC) and minimum bactericidal concentrations (MBC). Furthermore, an in vivo pharmacological study was carried out to assess the anti-inflammatory activity of the aqueous and ethanolic extracts of *S.costus*.

## 2. Results

### 2.1. Antilithiatic Activity In Vitro Study

#### 2.1.1. In Vitro Study of Struvite Crystallization

The microscopic crystallization of struvite crystals is expressed in mm^3^ using an optical microscope, in the presence of various concentrations of aqueous and ethanolic extracts of the studied plantas well as the cystone solution which served as a positive control. Table 1 shows that the number of crystals decreases along with concentration increasing of the extracts. 

**Figure 2:**
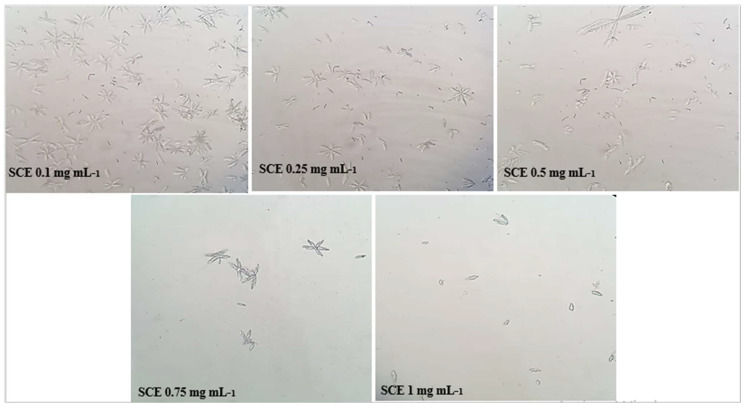
Microscopic observation of struvite crystals at different concentrations of ethanolic extracts of *S. costus*.

**Figure 3:**
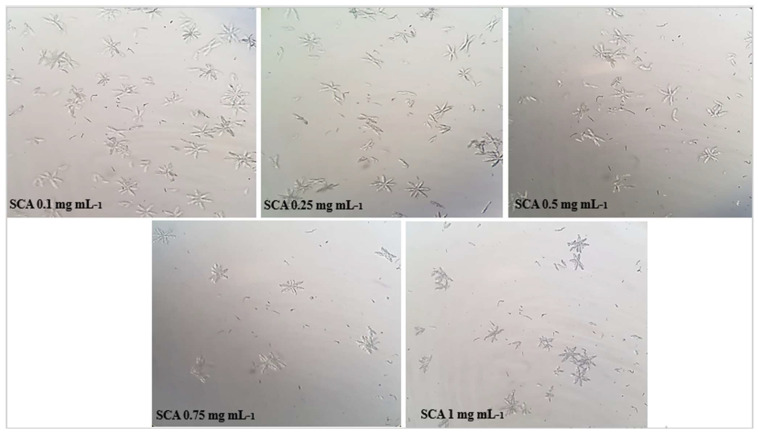
Microscopic observation of struvite crystals at different concentrations of aqueous extracts of *S. costus*.

**Figure 4:**
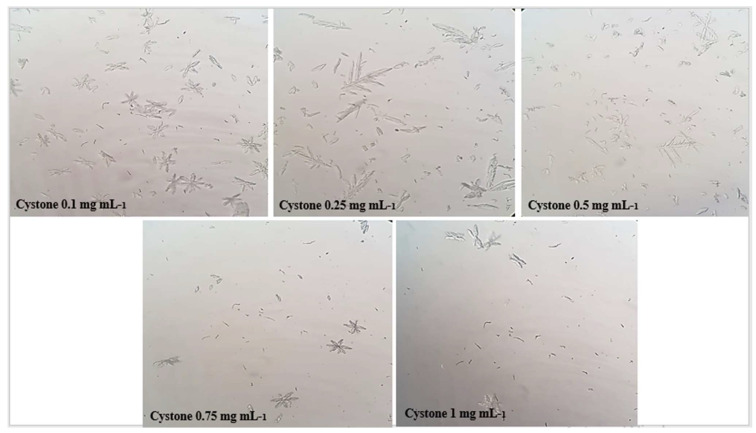
Microscopic observation of struvite crystals at different concentrations of cystone.

The results obtained in Figure 2, Figure 3 and Figure 4 show that the number of struvite crystals was reduced following the addition of the extracts of the plant under study to the crystals. It is also observed that there is a change in the morphology of the crystals at the concentration of 1 mg mL^−1^ of ethanolic extract, and the size of the crystals becomes smaller at the concentration of 0.75 mg mL^−1^ and 1 mg mL^−1^ of all solutions. The number of crystals between the concentrations of 0.1 mg mL^−1^ and 1 mg/mL for ethanolic extract decreases from (>1000/mm^3^ to <100/mm^3^) and for the aqueous extract decreases from (>1000 mm^3^ to 700 mm^3^), using a light microscope (400×).

#### 2.1.2. Turbidity Inhibition Test

**Figure 5:**
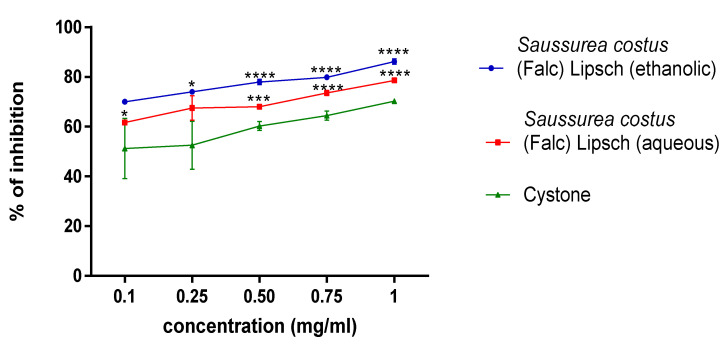
Effect of aqueous and ethanolic extracts from *S. costus* on struvite crystallization. (Each value represents the average of three trials ± SD). * *p* value < 0.05, *** *p* value < 0.005, **** *p* value < 0.001.

The percentage inhibition of struvite crystals of the aqueous and ethanolic extracts of the plant studied at different concentrations (0.1; 0.25; 0.5; 0.75; 1 mg mL^−1^) was determined using the turbidimetric method with cystone as a positive control. Data analysis from the curves in Figure 5 revealed that the ethanolic extract of *S. costus* inhibited struvite crystals more effectively than the aqueous extract of the same plant. In addition, the highest crystal inhibition ratio was (87.447 ± 1.107) (*p* < 0.001) and (78.565 ± 0.422) (*p* < 0.001) at a 1 mg/mL concentration for ethanolic and aqueous extracts of *S. costus*, respectively.

#### 2.1.3. Characterization of the Crystals by FT-IR

**Figure 6:**
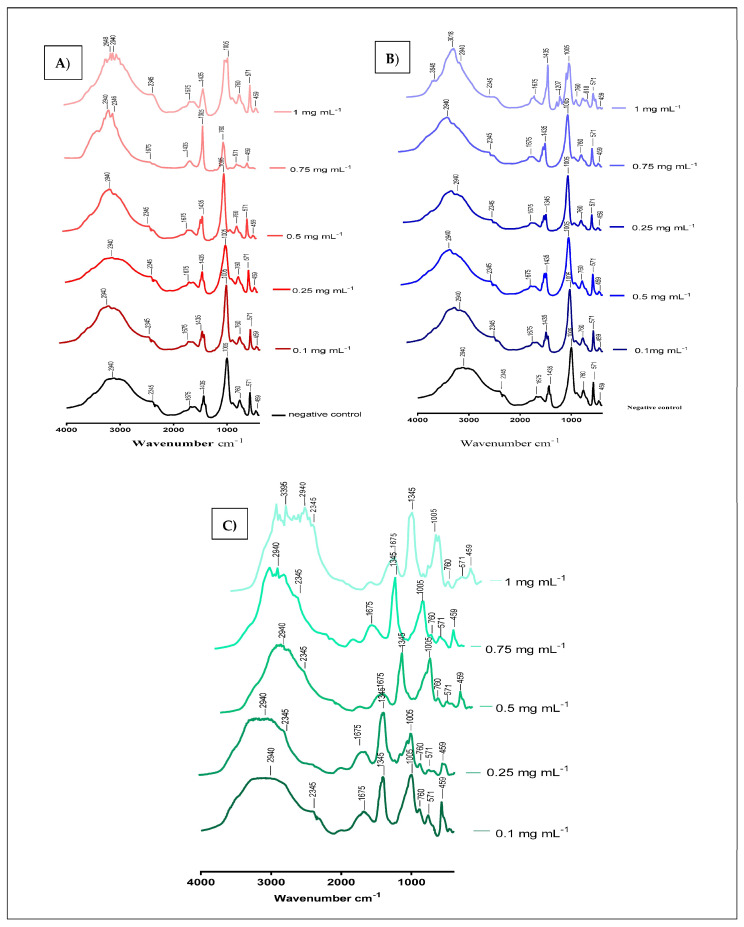
(**A**) FT-IR spectra of struvite before and after exposure to various concentrations of *S. costus* ethanolic extracts. (**B**) FT-IR spectra of struvite before and after exposure to various concentrations of *S. costus* aqueous extracts of. (**C**) FT-IR spectra of struvite before and after exposure to cystone at various concentrations.

The chemical composition of the crystals synthesized in the turbidity experiment, in the absence and presence of different concentrations (0.1; 0.25; 0.5; 0.75; 1 mg mL^−1^) of aqueous and ethanolic extracts of *S. costus* was determined by infrared spectroscopy, is presented in Figure 6. These results indicate that the synthesized crystals are composed of struvite, as struvite is characterized by the presence of the peaks 2345 cm^−1^ and 1435 cm^−1^ (Table 2). The variation in band intensity between concentrations can be explained by the interaction between the molecules in the extracts and the crystal structure of struvite.

### 2.2. Antimicrobial Activity

#### 2.2.1. Qualitative Evaluation

##### Antibiotic Susceptibility Test

The diameters of the zones of bacterial growth inhibition for the strains studied are presented in Table 3. They also demonstrate the sensitivity of the strains to antibiotics. For example, *Klebsiella pneumoniae* is sensitive to three antibiotics (Ofloxacin, Norfloxacin, and Cefotaxime), *Staphylococcus aureus* is only sensitive to Ofloxacin, and both *Pseudomonas aeruginosa* and *Escherichia coli* are resistant to antibiotics.

##### Disk Diffusion Test

The antibacterial activity of *S. costus* extracts on four bacterial strains was tested using the disc diffusion method. The obtained inhibition zones demonstrate that the ethanolic extract of the studied plant has antimicrobial activity against the bacterial strains. In addition, the ethanolic extract of 200 mg mL^−1^ and 400 mg mL^−1^ shows an effect on *Klebsiella pneumoniae* and *Staphylococcus aureus* strains, both of which are antibiotic resistant, which shows a sensitivity to ethanolic extract with inhibition zone values ranging from 12.01 mm to 14.00 mm, and the 200 mg mL^−1^ and mg mL^−1^ aqueous extracts show an effect on a single strain of *Staphylococcus aureus,* with an inhibition zone of 8.01 and 9.02 respectively (Table 4).

#### 2.2.2. Quantitative Evaluation

The determination of MIC and MBC inhibition parameters allows us to not only confirm, quantify and compare activities, but also to characterize the nature of the effect revealed by an extract on a given microorganism.

##### Minimum Inhibitory Concentration (MIC) and Minimum Bactericidal Concentration (MBC)

Table 5 shows that the ethanolic extract of *S. costus* inhibits *Staphylococcus aureus* (Gram+) at an MIC of 50 mg mL^−1^ and inactivates *Klebsiella pneumoniae* (Gram−) at an MIC of 200 mg mL^−1^, and the MIC of the aqueous extract of *S. costus* (Falc) Lipsch against *Staphylococcus aureus* and *Klebsiella pneumoniae* is 400 mg mL^−1^ and 200 mg mL^−1^, respectively. In addition, the results obtained in Table 5 show that the ethanolic extract of *S. costus* has a bactericidal effect for *Staphylococcus aureus* (Gram+) with an MBC detected at 100 mg mL^−1^, and has a bacteriostatic effect for *Klebsiella pneumoniae* (Gram−), with a bacterial growth observed in all the concentrations at or above the MIC. These results also show that the aqueous extract of *S. costus* has a bacteriostatic effect for *Staphylococcus aureus* (Gram+) and *Klebsiella pneumoniae* (Gram−) with a bacterial growth observed in all the concentrations at or above the MIC.

### 2.3. Anti-Inflammatory Activity

#### The Carrageenan Edema Test

**Figure 7:**
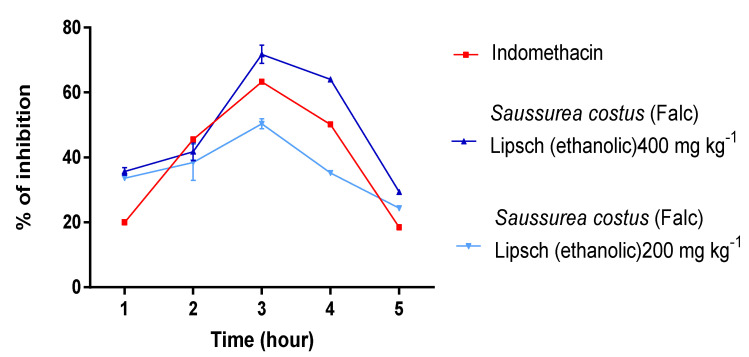

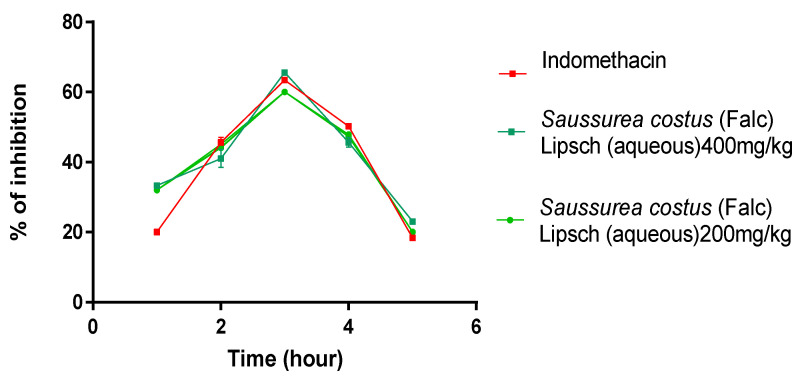
Effect of ethanolic and aqueous extracts of *S. costus* on carrageenan-induced inflammatory edema of the left hind paw in rats.

The obtained data for the influence of the *S. costus* extracts (aqueous) on carrageenan-induced edema are displayed in Figure 7. Both extracts (ethanolic and aqueous) of *S. costus* at 200 and 400 mg kg^−1^, administered orally, showed significant (*p* < 0.05) anti-inflammatory activity compared to the standard indomethacin.

The ethanolic extract of *Saussurea costus* showed a maximum reduction and inhibition of edema by 60% and 66% at 200 and 400 mg kg^−1^ respectively, as compared to aqueous extracts, which showed a maximum reduction and inhibition of edema by 52.04% and 64.02% at 200 and 400 mg kg^−1^ and close to standard indomethacin at the dose of (63.02%) during the same phases.

## 3. Discussion

Figure 1 shows the GC-MS analysis; our previous work [20] on the same extracts revealed that they were composed of the following: caryophyllene oxide, 1,3-propanediol, 2-(hydroxymethyl)-2-nitro, dihydrodehydrocostus lactone, quercetin, cyclodecacyclotetradecene and dehydrocostuslactone for the ethanolic extract of *S. costus*; 9,12,15-octadecatrienoic acid,(z,z),alpha guaiene,2(3H)-benzofuranone,6-ethenythexhydroxy, Cholest-7-en-3-ol,4-methyl-,3.beta,4.alpha and cyclodecacyclotetradecene,14,15-didehydroxy for the aqueous extract of *S. costus*.

Microscopic crystallization of struvite, as shown in Table 1 and Figure 2, Figure 3 and Figure 4, indicated that the number of crystals were decreased upon increasing the concentration of the studied extracts. Our data also revealed that the *S. costus* ethanolic extract was effective against struvite and can also suppressed the ammonium–magnesium phosphate crystals formation, since the number of crystals remaining at a concentration of 1 mg mL^−1^ is 100/mm^3^; thus, these results are similar to those of Sadki et al. [21]. Their research revealed that the studied plant is effective, with the number of crystals reaching 100/mm^3^ at a concentration of 1 mg mL^−1^.

The results displayed in (Figure 5) indicated that the *S. costus* ethanolic extract of was able to treat struvite crystals and had a significant effect with an inhibition percentage f (87.447 ± 1.107) (*p* < 0.001) at the concentration of 1 mg mL^−1^. Regarding the turbidimetric test results of the synthesized struvite crystals, the chemical composition of the crystals was identified using the Fourier transform infrared spectroscopy (FT-IR) technique. The analysis of FT-IR spectra showed (Figure 6) the presence of a band at 2940 cm^−1^, attributed to the antisymmetric stretching band of ammonium (vs. (NH_4_)) and the symmetric stretching band of amines (vs. (N-H)) observed at 1675 cm^−1^. The P=O stretching absorption bands were observed at 760 cm^−1^, the PO_4_ absorption band was observed at 1005 cm^−1^, the antisymmetric stretching band of primary amines (vas (PO_4_)) was observed at 571 cm^−1^, and the symmetric absorption band (vas(H-O-H)) was observed at 2345 cm^−1^ [22,23]. The FT-IR spectrum analysis proved that the compound type is struvite for the negative control. Moreover, the different infrared spectra obtained when processing the crystals at different concentrations show the same bands as the negative control but with a change in the intensity of the peaks, which shows that the active molecules present in these extracts have an effect that can deform or degrade the bonds of the struvite atoms, and this is also shown in Figure 2, Figure 3 and Figure 4, where it was observed that when the concentration of plants increases, the number of crystals decreases, their size changes, and they break.

Regarding the antimicrobial activity and according to Table 3, the inhibition zones of the studied extracts show that the ethanolic extract of *S. costus* has the best activity on two strains (*Staphylococcus aureus* and *Klebsiella pneumoniae*) among all the tested strains with the inhibition zones of 14.00 mm and 12.04 mm, respectively, while the aqueous extract of the studied plant has low activity on the same strains. The inhibitory potential of the extracts was confirmed by a quantitative MIC test, and the results in Table 5 show that the ethanolic extract of *S. costus* inhibits the growth of *Staphylococcus aureus* and *Klebsiella pneumoniae* strains with MICs of 50 mg mL^−1^ and 200 mg mL^−1^ respectively, According to this study, all the extracts of *S. costus* have a bacteriostatic effect, except for the ethanolic extract, which has a bactericidal effect with a significant MBC (100 mg mL^−1^) on the *Staphylococcus aureus* strain. From these results, we can conclude that the microbial efficacy of the ethanolic extract of the studied plant is probably due to the presence of the flavonoid compound. Similar results on the efficacy of *S. costus* extracts were reported by Sughra et al. [24] and AL-Kattan et al. [25]. These authors showed that *S. costus* extract was active on *Staphylococcus aureus* and *Klebsiella pneumoniae* strains.

With regard to *in vivo* pharmacological activity, carrageenan-induced rat paw edema is known to be sensitive to cyclooxygenase inhibitors and has been used to evaluate the effect of nonsteroidal anti-inflammatory agents, which primarily inhibit cyclooxygenase involved in prostaglandin synthesis [26].

The anti-inflammatory activity of *S. costus* extracts presented in Figure 7 showed that ethanolic extracts resulted in maximum reduction and the inhibition of edema by 60% and 66% at 200 and 400 mg kg^−1^ (at 3 h) respectively, compared to aqueous extracts with 52.04% and 64.02% at 200 and 400 mg kg^−1^ (at 3 h) respectively, which is similar to standard indomethacin at 10 mg kg^−1^ (63.02%) during the same phases. The results we obtained are similar to those of Gokhale et al. [27], who demonstrated the inhibitory efficacy of extracts of *S. costus* (Falc) Lipsch, causing the maximum inhibition of edema of 42% and 67%, at doses of 200 mg kg^−1^ and 400 mg kg^−1^ (at 3 h). The efficacy of these extracts is probably due to the presence of secondary metabolic compounds in these extracts such as phenolic terpenoids, flavonoids, tannins, etc., and this is in agreement with many studies in the literature reporting that many plants containing these chemical compounds are known to possess potent anti-inflammatory properties that act through the inhibition of prostaglandin pathways [28].

## 4. Materials and Methods

### 4.1. Plant Material

The roots of *S.costus* were collected in the Himalayan region, in Jammu (52°15′ N, 7°06′ W) and exported to Morocco on 15 March 2021. This plant was authenticated at the Faculty of Medicine and Pharmacy, Dental Medicine, University Sidi Mohammed Ben Abdellah of Fez, Morocco (voucher specimen n° LERH-SC/15-03-21) [19].

### 4.2. Extraction

The extraction was performed according to the Soxhlet method [29,30]. A total of 10 g of ground *S. costus* was placed in a cartridge and introduced into a Soxhlet extractor, which has a 250 mL flask at the bottom, into which 150 mL of solvent (70% ethanol or 100% water) was introduced. The solvent was boiled in the flask, and its vapor passed through the side tube and condensed to a refrigerated level. The filtered extracts were then evaporated under vacuum using a rotary evaporator. The ethanolic extract had an extraction yield of 18.79 ± 0.01 and the aqueous extract had an extraction yield of 28.41± 0.01 [19].

### 4.3. Antilithiatic Activity In Vitro Study

#### 4.3.1. In Vitro Study of Struvite Crystallization

The purpose of this experiment was to see how extracts of the plant *S. costus* affected struvite crystals in the presence and absence of extracts. This experiment was performed using an in vitro struvite crystallization protocol described by C. Sadki et al. [21,31] with some modifications. This protocol involves the preparation of two solutions: solution A, which consists of 0.1 M potassium dihydrogen phosphate (KH_2_PO_4_), and solution Bm which consists of magnesium chloride (41 g), ammonium chloride (50 g) and 20 mL ammonium hydroxide diluted 10 fold in 50 mL of bi-distilled water. A total of 1 mL of solution A was poured into glass tubes, followed by 1 mL of extract at different concentrations (0.1, 0.25, 0.5, 0.75 and 1 in mg mL^−1^). Instead of the extract, the negative control tubes contained 1 mL of distilled water, and the positive control tubes contained 1 mL of cystone at different concentrations (0.1, 0.25, 0.5, 0.75, and 1 mg mL^−1^). Then, 1 mL of solution B was added, and the tubes were incubated at 37 °C for 30 min. The morphology and number of struvite crystals in each sample were then determined using an OLYMPUS U-SPT Japan microscope (500×) [21].

#### 4.3.2. Turbidity Inhibition Test

The turbidimetric method was used to evaluate the anti-ammonium–magnesium phosphate activity of struvite crystals that we synthesized in Section 4.3.1 (in vitro study of struvite crystallization). The inhibition of struvite crystallization was studied by measuring the optical density of the solutions prepared in the absence and presence of the aqueous and ethanolic extracts of *S. costus* in a LABTRON LUS-Series, UK, double-beam UV/Vis spectrophotometer, and the optical density was determined at λ = 620 nm [32,33]. The percentage of inhibition (%) of struvite crystals produced by the plant extracts studied was calculated using the following formula [19]:

(1) $$\%\;\mathrm{Of}\;\mathrm{inhibition}\;=\frac{Control\;absorbance-Test\;absorbance\;}{Cpntrol\;absorbance}$$

Absorbance of control = Absorbance without inhibitor.

Absorbance of test = Absorbance in presence of inhibitor.

#### 4.3.3. Characterization of the Crystals

Fourier transform infrared spectroscopy was used to characterize the crystals synthesized according to the Section (4.3.1). A total of 1 mL of solutions A((KH_2_PO_4_) 0.1 M) and 1 mL of solution B ((MgCl_2_ (41 g), NH_4_Cl (50 g), and 20 mL of NH_4_OH (diluted 10-fold) in the presence and absence of 1 mL of *S. costus* aqueous and ethanolic extracts and the cystone (positive control ) were stirred continuously for 30 min and stored at 37 °C for 24 h. The precipitate that formed was then dried before examination. This was analyzed by dissolving 5% of the precipitate in 95% KBr [34]. The resulting homogeneous powder was placed in a 13 mm diameter pellet mold. For 3 min, the mold was subjected to a pressure of 10 tons created by a vacuum press, after which translucent pellets of 1 mm thickness were obtained and analyzed in an FT-IR instrument (Burker Optic GMBH, Co. KG., Ettlingen, Germany). The analysis was achieved over a wide range of wavelengths (4000 and 400 cm^−1^).

### 4.4. Antimicrobial Activity

#### 4.4.1. Tested Bacterial Strains

The bacterial strains tested were previously isolated from the environment of the hemodialysis center of the Hassan II University Hospital (Fez). They are *Staphylococcus aureus*, which is a Gram-positive cocci, while *Pseudomonas aeruginosa*, *Escherichia coli* and *Klebsiella pneumoniae* are Gram-negative bacilli.

#### 4.4.2. Antibiotic Susceptibility Test

The susceptibility of the strains to antibiotics was investigated using the disc diffusion method [35], for the classification of bacterial strains into susceptible, intermediate, and resistant categories. The resistance (R) to antibiotics was defined by the Antibiogram Committee of the Microbiology French Society, 2019 edition [36]. The antibiotics used in our study are (E) Erythromycin; (OFX) Ofloxacin; (TIC) Ticarcillin; (OX) Oxacillin; (AMP) Ampicillin; (NOR) Norfloxacin; (CAZ) Ceftazidime; (CTX) Cefotaxime.

#### 4.4.3. Qualitative Evaluation

##### Disc-Diffusion Method

The antimicrobial effect of *S. costus* extracts was determined by the sterile disk diffusion method; this method consists of using sterile filter paper disks, 6 mm in diameter and impregnated with aqueous and ethanolic extracts of the plant studied, notably with (200 mg mL^−1^ and 400 mg mL^−1^), at a rate of 5 μL/disk. These disks are placed on the surface of an agar medium (Muller-Hinton), previously inoculated with a bacterial suspension, The disks are left 15 to 30 min at room temperature, then incubated at 37 °C for 24 h [36,37].

#### 4.4.4. Quantitative Evaluation

##### Minimum Inhibitory Concentration (MIC)

The MIC was defined as the lowest concentration of antibiotic required to inhibit the growth of a bacterium. In this experiment, we determined the MIC of extracts that tested positive in the first test (disk diffusion test). We used a 96-well plate in which each well contained 20 μL of pure water + 20 μL of strain + 20 μL of aqueous and ethanolic extracts of (400 to 0.39 mg mL^−1^) + 140 μL of BHI (Brain heart infusion) medium. A triphenyl tetrazolium chloride (TTC) indicator revealed bacterial growth after 24 h of incubation at 37 °C [38,39].

##### Minimum Bactericidal Concentration (MBC)

The MBC is the minimum bactericidal concentration, which is defined as the lowest concentration of antimicrobial agent required to kill 99.9% of the final inoculum after 24 h of incubation at 37 °C. Depending on the MIC, the MBC can be determined after microdilution. Extracts from wells with concentrations at or below the MIC are spread on nutrient agar and after 24 h of incubation at 37 °C, bacterial growth was observed [40].

### 4.5. In Vivo Study

#### 4.5.1. Anti-Inflammatory Activity

##### Animal Material

The Wistar rats were provided by the animal house of the Faculty of Medicine and Pharmacy, Dental Medicine Fez. Before being included in the experiment, they were randomly divided into 5 groups in conventional cages and were given a 15 day adaptation period with free access to food and water, a controlled temperature of 28 ± 2 °C, a humidity of 60 and a light/dark cycle of 12/12 h.

##### The Carrageenan Edema Test

The evaluation of the anti-inflammatory activity of the aqueous and ethanolic extracts of the plant studied was carried out by a chemical method with carrageenan [40], Before the injection of 1% carrageenan (10 mg Kg^−1^) to all the animals in the right posterior leg, the rats were divided into different groups (n = 6) (180–200 g); each group received, orally, the following experimental solutions:

Positive control group (n = 6): the reference drug indomethacin 10 mg Kg^−1^.

Group A Dose 1 (n = 6): aqueous extract of *S. costus* (200 mg Kg^−1^).

Group B Dose 2 (n = 6): aqueous extract of *S. costus* (400 mg Kg^−1^).

Group C Dose 1 (n = 6): ethanolic extract of *S. costus* (200 mg Kg^−1^).

Group D Dose 2 (n = 6): ethanolic extract of *S. costus* (400 mg Kg^−1^).

The left hind paw was not treated and was considered a negative control. The difference in volume between the left and right paws was measured using a 7500 digital plethysmometer at (1 h, 2 h, 3 h, 4 h, and 5 h) after induction of inflammation. The following formula was used to calculate the percent inhibition (%) of inflammation:

(2) $$\mathrm{Of}\;\mathrm{inhibition}\;(\%)=\frac{\mathrm{mean}\left(\mathrm{Vr}-\mathrm{Vl}\right)\mathrm{Control}-\mathrm{mean}\left(\mathrm{Vr}-\mathrm{Vl}\right)\mathrm{treated}}{\mathrm{mean}\left(\mathrm{Vr}-\mathrm{Vl}\right)\mathrm{Control}}\times 100$$

Vr: the volume of the edema on the right hind leg.

Vl: the volume of the edema on the left hind leg.

### 4.6. Data Analysis

The data are presented as the means of three independent (triplicate) experiments and analyzed with a one-way ANOVA. (*p* < 0.05) values were considered significant. GraphPad Prism 7 was used for statistical analysis.

## 5. Conclusions

The efficacy of *S. costus* (Falc) Lipsch extracts, especially the ethanolic extract, plays a key role in the prevention and treatment of struvite crystal formation. The tested extracts of *Saussurea costus* also showed interesting antimicrobial properties, the ethanolic extract being considered the most important antibiotic to inhibit the bacteria responsible for struvite crystal formation. On the other hand, the anti-inflammatory study on rats showed the efficacy of these extracts, the results being quite close between the extracts, and we conclude that the plant of *Saussurea costus* has an important role in relieving the type of pain associated with bacterial infection. Therefore, we believe that it is important in future research to study the effect of the ethanolic extract of the plant in the treatment of struvite in vivo.

## Figures and Tables

**Table 1:** The number of struvite crystals in the presence of extracts of plant and cystone solution.

Concentrations	Cystone Solution	*S. costus* (Aqueous)	*S. costus* (Ethanolic)
0.1 mg mL^−1^	>1000/mm^3^	>1000/mm^3^	>1000/mm^3^
0.25 mg mL^−1^	>700/mm^3^	>900/mm^3^	>900/mm^3^
0.5 mg mL^−1^	>600/mm^3^	>800/mm^3^	>600/mm^3^
0.75 mg mL^−1^	500/mm^3^	>700/mm^3^	500/mm^3^
1 mg mL^−1^	400/mm^3^	700/mm^3^	<100/mm^3^

**Table 2:** Absorption peaks for the struvite crystals.

Bonds	Absorption Peaks
vas (NH4)	2940 cm^−1^
vs (N-H)	1675 cm^−1^
P=O	760 cm^−1^
PO_4_	1005 cm^−1^
vas (PO_4_)	571 cm^−1^
vas (H-O-H)	2345 cm^−1^

**Table 3:** Antibiotic susceptibility test of the bacterial strains studied.

	Bacteria	*Staphylococcus aureus*	*Pseudomonas aeruginosa*	*Escherichia coli*	*Klebsiella pneumoniae*
Antibiotic					
**Erythromycine**		-	R (7 mm)	R (6 mm)	R (6 mm)
**Ofloxacine**		S (26 mm)	R (8 mm)	R (6 mm)	S (27 mm)
**Oxacilline**		R (6 mm)	R (6 mm)	R (6 mm)	R (6 mm)
**Ampicilline**		-	R (6 mm)	R (6 mm)	R (6 mm)
**Norfloxacine**		-	R (12 mm)	R (6 mm)	S (25 mm)
**Ceftazidime**		R (14 mm)	R (6 mm)	R (6 mm)	R (15 mm)
**Cefotaxime**		R (18 mm)	R (6 mm)	R (6 mm)	S (20 mm)

-: The antibiotic does not correspond to this strain, S: sensitive, R: resistant.

**Table 4:** Inhibition diameters of aqueous and ethanolic extracts of *S. costus*. (Each value represents the average of three trials ± SD).

Extracts	*Staphylococcus aureus* (mm)	*Pseudomonas* *aeruginosa* (mm)	*Escherichia coli* (mm)	*Klebsiella pneumoniae* (mm)
*Saussurea costus* (Falc) Lipsch (aqueous) 400 mg mL^−1^ (2 mg/disc)	8.01 ± 0.01	-	-	-
*Saussurea costus* (Falc) Lipsch (aqueous) 200 mg mL^−1^ (1 mg/disc)	9.02 ± 0.03	-	-	-
*Saussurea costus* (Falc) Lipsch (ethanolic) 400 mg mL^−1^ (2 mg/disc)	14.00 ± 0.00	-	-	12.04 ± 0.04
*Saussurea costus* (Falc) Lipsch (ethanolic) 200 mg mL^−1^ (2 mg/disc)	12.03 ± 0.005	-	-	12 ± 0.00

**Table 5:** Minimum inhibitory concentration (MIC) and Minimum bactericidal concentration (MBC) of aqueous and ethanolic extracts of *S. costus* (expressed in mg mL^−1^).

	*S. costus* (Ethanolic)		*S. costus* (Aqueous)	
Strain	*Staphylococcus aureus* (Gram+)	*Klebsiella pneumoniae* (Gram−)	*Staphylococcus aureus* (Gram+)	*Klebsiella pneumoniae* (Gram−)
MIC	50 mg mL^−1^	200 mg mL^−1^	400 mg mL^−1^	200 mg mL^−1^
MBC	100 mg mL^−1^	-	-	-
Effect	Bactericidal	Bacteriostatic	Bacteriostatic	Bacteriostatic

## Data Availability

Not applicable.

## Sample Availability

Samples of the compounds are available from the authors.
